# Arterial Wall Inflammation and Increased Hematopoietic Activity in Patients With Primary Aldosteronism

**DOI:** 10.1210/clinem/dgz306

**Published:** 2019-12-25

**Authors:** Charlotte D C C van der Heijden, Esther M M Smeets, Erik H J G Aarntzen, Marlies P Noz, Houshang Monajemi, Simone Kersten, Charlotte Kaffa, Alexander Hoischen, Jaap Deinum, Leo A B Joosten, Mihai G Netea, Niels P Riksen

**Affiliations:** 1 Department of Internal Medicine, Radboud University Medical Center, Nijmegen, the Netherlands; 2 Radboud Institute for Molecular Life Sciences, Radboud University Medical Center, Nijmegen, the Netherlands; 3 Department of Nuclear Medicine, Radboud University Medical Center, Nijmegen, the Netherlands; 4 Department of Internal Medicine, Rijnstate Hospital, Arnhem, the Netherlands; 5 Department of Human Genetics, Radboud University Medical Center, Nijmegen, the Netherlands; 6 Centre for Molecular and Biomolecular Informatics, Radboud Institute for Molecular Life Sciences, Radboud University Medical Center, Nijmegen, the Netherlands; 7 Department of Medicine, University Hospital Dresden, Technische Universität, Dresden, Germany; 8 Department of Medical Genetics, Iuliu Hațieganu University of Medicine and Pharmacy, Cluj-Napoca, Romania; 9 Department for Genomics & Immunoregulation, Life and Medical Sciences 12 Institute, University of Bonn, Bonn, Germany

**Keywords:** primary aldosteronism, inflammation, atherosclerosis, immune system, mineralocorticoid, ^18^F-FDG PET-CT

## Abstract

**Context:**

Primary aldosteronism (PA) confers an increased risk of cardiovascular disease (CVD), independent of blood pressure. Animal models have shown that aldosterone accelerates atherosclerosis through proinflammatory changes in innate immune cells; human data are scarce.

**Objective:**

The objective of this article is to explore whether patients with PA have increased arterial wall inflammation, systemic inflammation, and reprogramming of monocytes.

**Design:**

A cross-sectional cohort study compared vascular inflammation on 2’-deoxy-2’-(18F)fluoro-D-glucose; (^18^F-FDG) positron emission tomography–computed tomography, systemic inflammation, and monocyte phenotypes and transcriptome between PA patients and controls.

**Setting:**

This study took place at Radboudumc and Rijnstate Hospital, the Netherlands.

**Patients:**

Fifteen patients with PA and 15 age-, sex-, and blood pressure-matched controls with essential hypertension (EHT) participated.

**Main Outcome Measures and Results:**

PA patients displayed a higher arterial ^18^F-FDG uptake in the descending and abdominal aorta (*P* < .01, *P* < .05) and carotid and iliac arteries (both *P* < .01). In addition, bone marrow uptake was higher in PA patients (*P* < .05). Although PA patients had a higher monocyte-to-lymphocyte ratio (*P* < .05), systemic inflammatory markers, cytokine production capacity, and transcriptome of circulating monocytes did not differ. Monocyte-derived macrophages from PA patients expressed more *TNFA*; monocyte-derived macrophages of healthy donors cultured in PA serum displayed increased interleukin-6 and tumor necrosis factor-α production.

**Conclusions:**

Because increased arterial wall inflammation is associated with accelerated atherogenesis and unstable plaques, this might importantly contribute to the increased CVD risk in PA patients. We did not observe inflammatory reprogramming of circulating monocytes. However, subtle inflammatory changes are present in the peripheral blood cell composition and monocyte transcriptome of PA patients, and in their monocyte-derived macrophages. Most likely, arterial inflammation in PA requires interaction between various cell types.

Primary aldosteronism (PA) is the most common cause of secondary hypertension, with an estimated prevalence of 5% in the hypertensive population (1). In patients with PA, autonomous adrenal overproduction of aldosterone increases renal sodium reabsorption, which subsequently induces hypertension. Interestingly, patients with PA have a higher risk for atherosclerotic cardiovascular events than patients with essential hypertension (EHT) with similar blood pressure levels, suggesting that chronic exposure to high aldosterone levels has additional direct detrimental effects on the cardiovascular system (2).

Cardiovascular diseases (CVD) result from atherosclerosis, a chronic low-grade inflammatory arterial wall disease that results from a complex interplay between the vascular endothelium, smooth muscle cells, and the immune system (3). Monocyte-derived macrophages are the most abundant immune cells in atherosclerotic plaques, driving plaque formation, progression, and destabilization (4). Interestingly, cells from the innate and the adaptive immune system express the mineralocorticoid receptor (MR) (5). We recently suggested that the proatherogenic effects of aldosterone could find its origin in activation of innate immune cells (6). Supporting this hypothesis, rodent models have shown that aldosterone accelerates the development of atherosclerotic plaques (7). We showed that short-term exposure of human monocytes to aldosterone in vitro induces a persistent immunological imprinting of monocytes, resulting in a long-lasting proinflammatory phenotype of monocyte-derived macrophages (8). This concept of innate immune reprogramming after transient exposure of monocytes to proinflammatory stimuli is called *trained immunity* and has been suggested to play a role in the chronic inflammation driving atherogenesis (9-12). Trained immunity can occur in circulating monocytes and their bone marrow progenitors (13). Therefore, the classical in vivo read-outs of a trained phenotype consist of increased myelopoiesis and a higher ex vivo cytokine production capacity, which are believed to result from an increased and long-lasting bone marrow mobilization of proinflammatory monocytes (14).

Human data on vascular wall inflammation and immune cell function of patients with PA are scarce (6). Ultrasound measurements were suggestive of preclinical atherosclerosis in PA patients (15, 16), but are unable to provide information on the inflammatory component of atherogenesis. In recent years 2’-deoxy-2’-(^18^F)fluoro-D-glucose positron emission tomography with computed tomography (^18^F-FDG PET-CT) imaging has emerged as a tool to assess arterial wall inflammation. ^18^F-FDG uptake in the arterial wall reflects local inflammation, correlates with high-risk plaque features on CT and magnetic resonance imaging, and correlates with macrophage numbers in atherosclerotic plaques (17). Additionally, in patients with atherosclerosis, ^18^F-FDG uptake in bone marrow was increased and corresponded to an increased progenitor potential (18).

In the present study, we investigated whether patients with PA have enhanced vascular wall inflammation and increased hematopoietic activity on PET-CT, and comprehensively assessed the inflammatory profile and immune cell function of these patients to explore the hypothesis that chronic inflammation contributes to the development of CVD and is mediated by persistent activation of monocytes.

## Methods

### Participants

We included 15 patients with PA and 15 matched controls with EHT (Table 1) between February 2017 and September 2018, from the Radboudumc, (Nijmegen, the Netherlands) and the Rijnstate Hospital (Arnhem, the Netherlands). In EHT controls, secondary hypertension including PA was ruled out (baseline serum aldosterone < 0.42 mmol/L and an aldosterone-to-renin ratio value of < 0.09 nmol/mU or a negative salt-loading test [aldosterone < 0.14 nmol/L after saline infusion]). Aldosterone levels in plasma and renin levels in serum were obtained following a standardized protocol with venipuncture performed between 8 am and 10 am after a minimum of 5 minutes of absolute rest in the supine position. Plasma renin and serum aldosterone concentrations were measured by the Department of Laboratory Medicine of the Radboudumc. Plasma renin concentrations were measured by immunoradiometric assay (RENIN III generation, CIS Bio International), and serum aldosterone concentrations were measured after extraction and paper chromatography with recovery correction (19). In all patients PA was confirmed according to current guidelines with a salt-loading test (circulating aldosterone concentrations > 0.28 nmol/L after intravenous infusion of 2 L saline in 4 hours). Exclusion criteria included clinically manifest CVD (a history of transient ischemic attacks, cerebrovascular accidents, myocardial infarction, pectoral angina, and peripheral artery disease), diabetes mellitus, smoking, and inflammatory or autoimmune diseases, or the use of immunosuppressive drugs such as corticosteroids and nonsteroidal anti-inflammatory drugs. In all participants, MR antagonists were discontinued for a minimum of 4 weeks. Diuretics, angiotensin-converting enzyme inhibitors, and β-blockers were discontinued for a minimum of 10 days. Adequate blood pressure control was obtained by the use of calcium antagonists or doxazosine both for PA patients and EHT control patients. Statins were used by 1 PA patient and 1 EHT control and were discontinued for 1 week before the study day. No participants used antiplatelet drugs or oral anticoagulants.

**Table 1.** Baseline characteristics

Clinical Characteristics	EHT Controls (n = 15)	PA Patients (n = 15)	*P*
Sex: male (%)	7 (47)	7 (47)	*P = *1.00
Age (years)	47 (25-74)	50 (35-71)	*P *= .55
BMI (kg/m^2^)	25.6 (3.5)	25.8 (3.3)	*P *= .82
Systolic BP (mm Hg)	146 (14)	155 (16)	*P *= .14
Diastolic BP (mm Hg)	83 (10)	90 (11)	*P *= .08
Fasting glucose, capillary	5.2 (0. 6)	5.2 (0.6)	*P *= .60
Smoker; past (%)	3 (20%)	2 (13%)	*P *= .62
Aldosterone (nmol/L)	0.31 (0.07-0.51)	0.97 (0.55-1.55)	*P < *10^–7^
Renin (nmol/L)	20 (4-56)	8 (< 3-23)	*P = *.08
Total cholesterol (mmol/L)	5.33 (0.94)	5.11 (0.94)	*P = *.53
HDL cholesterol (mmol/L)	1.38 (0.39)	1.34 (0.47)	*P *= .81
LDL cholesterol (mmol/L)	3.41 (0.81)	3.04 (0.71)	*P = *.20
Triglycerides (mmol/L)	1.24 (0.76-2.27)	1.58 (0.51-4.82)	*P *= .68
Non-HDL cholesterol (mmol/L)	3.97 (0.91)	3.78 (0.86)	*P *= .56

Abbreviations: BMI, body mass index; BP, blood pressure; EHT, essential hypertension; HDL, high-density lipoprotein; LDL, low-density lipoprotein; PA, primary aldosteronism.

The study and radiation exposure was approved by the regional institutional review board CMO Arnhem-Nijmegen (NL58835.091.16). For the additional in vitro experiments, we isolated human peripheral blood mononuclear cells (PBMCs) from anonymous healthy volunteers (Sanquin Bloodbank). Each individual provided written informed consent before participation in this study. The study was conducted according to the principles of the International Conference on Harmonization–Good Clinical Practice guidelines.

### Anthropometric measurements

Blood pressure and heart rate were measured in the supine position in 4 repeated measures with a 5-minute interval for both arms after at least 10 minutes of rest; the average of the last 3 measures was used as the end point. All measurements were performed between 8 am and 10:30 am in a fasted state by a single investigator.

### Blood sampling and cell counts

Venous blood was drawn from the brachial vein after 24 hours of a low-carbohydrate diet and overnight fasting, between 8 am and 10:30 am. Serum and plasma was collected and stored at −80°C until assayed. Levels of total cholesterol, high-density lipoprotein cholesterol, and triglycerides were determined using an in-house analyzer (Cobas 8000; Roche Diagnostics), and low-density lipoprotein cholesterol was calculated with the Friedewald formula. Cell counts were obtained in fresh EDTA blood with a Sysmex automated hematology analyzer (XN-450; Sysmex Corporation).

### Positron emission tomography–computed tomography imaging

PET-CT scans were performed after a 24-hour low-carbohydrate diet and overnight fasting before infusion of ^18^F-FDG (2 MBq/kg) on a Siemens Biograph 40 mCT (Siemens Medical Solutions). After 120 minutes, following the European Association of Nuclear Medicine guidelines (17), participants underwent PET imaging and a low-dose noncontrast CT. Images were reconstructed according to EARL (EANM Research Ltd) protocols; a TrueX algorithm with point-spread function and time-of-flight measurements was constructed using 3 iterations, 21 subsets, matrix size 200 × 200 (pixel spacing of 4.07 mm), full width half maximum of 3 mm, and using 2 minutes of PET data. Postprocessing was performed using a 3-dimensional Gaussian filter kernel, 3.0 mm, using the Inveon Research Workspace 4.2 (Preclinical Solutions, Siemens Medical Solutions USA). The standardized uptake value (SUV_mean_) and SUV_max_ were extracted using the PyRadiomics toolbox (20). ^18^F-FDG uptake was assessed in 8 regions of interest (ROIs) by a single investigator (C.H.): the carotid arteries, the wall of the ascending descending and abdominal aorta, the iliac arteries, the bone marrow (L2-L3), spleen, and liver. For each ROI, we measured the SUV_mean_ and SUV_max_. For the left and right carotid artery, vertebrae L2 and L3, and the left and right iliac artery, the SUVs of both regions were averaged. The target-to-background ratio (TBR) for vascular ROIs was used as the primary outcome, following the recommendation of the European Association of Nuclear Medicine (17). The TBR was calculated from the ratio of the arterial SUV and background activity in the low-thoracic aortic blood pool. To avoid spillover from atherosclerotic lesions that might overestimate background blood pool activity, the background ROI was visually placed within the aortic lumen avoiding inclusion of the arterial wall, in a nondiseased segment. For the hematopoietic ROIs, TBR was calculated as the ratio of the splenic or bone marrow SUV and the mean background activity in the liver. Liver ^18^F-FDG uptake is expressed as SUV_raw_.

### Flow cytometry

Monocyte subpopulations were identified with flow cytometry using the lysis-no-wash strategy (BD Pharm Lyse lysing buffer, Becton Dickinson) on fresh EDTA blood. A total of 100 μl of EDTA blood was stained by monoclonal antibodies (CD16 FITC NKP15 Becton Dickinson, CD14 PE RMO52 Beckman Coulter, HLA-DR Immu357 PC5.5 Beckman Coulter, and CD45 PC7 J33 Beckman Coulter). Surface expression was assessed using FC500 and CytoFLEX flow cytometer (Beckman Coulter) and analyzed with Kaluza software version 2.1 (Beckman Coulter). The applied gating strategy was in short; monocytes were selected in the SSC/CD45+ plot, gated to SSC/HLA-DR + plot, identifying monocytes as CD45+ HLA-DR + cells with monocyte scatter properties. Exclusion of lymphocytes, and natural killer cells was performed by excluding CD45+ HLA-DR+ CD14– CD16– cells. In the CD14/CD16 plot, the percentages of gated monocyte subsets (classical CD14++CD16−), intermediate (CD14++CD16+), and nonclassical monocytes (CD14+CD16++) were used for analyses. Identification of monocytes subsets follows current recommendations (21).

### Peripheral blood mononuclear cells and monocyte isolation, processing, and stimulation

We isolated PBMC from EDTA blood using Ficoll-Paque density gradient centrifugation (GE Healthcare, 17-1440-03), after which we isolated CD14^pos^ monocytes using magnetic activated cell sorting (MACS, Miltenyi Biotec, 130-050-201). PBMCs were seeded in 96-well, round-bottom plates at 500 000 cells per well in RPMI (Roswell Park Memorial Institute) 1640 Dutch-modified culture medium (Life Technologies, 22 409 031) supplemented with 10 mM glutamine (Life Technologies, 35 050 087), 10 μg/mL gentamicin (Centraform), and 10 mM pyruvate (Life Technologies, 11 360 088) for 24 hours or 7 days. PBMCs were stimulated for 24 hours with 10 ng/mL *E coli* lipopolysaccharide (LPS, Sigma-Aldrich) (Toll-like receptor 4 [TLR4] ligand) or 10 µg/mL Pam3Cys (EMC microcollections, L2000) (Toll-like receptor 2 [TLR2] ligand) to induce monocyte-derived cytokine production (tumor necrosis factor-α [TNF-α], interleukin [IL]-1β, IL-6, IL-10, IL-1 receptor antagonist, IL-10), and for 7 days with 10 µg/mL phytohemagglutinin (PHA, Sigma, L9017) or 1 × 10^6^/mL heat-killed *Candida albicans—*both with 10% human serum—to induce lymphocyte-derived cytokine production (interferon-γ [IFNγ], IL-17, IL-22).

For assessment of monocyte-derived macrophages obtained from patients and controls, PBMCs obtained from every participant were seeded in flat-bottom well plates, adhered, and washed with warm phosphate-buffered saline for 3 washes. The remaining adherent monocytes were differentiated to macrophages in the presence of 10% of the serum obtained from the same study participant. On day 6, the monocyte-derived macrophages were stored in TRIzol (Fisher Scientific, 12 034 977) before, and after 4 hours of stimulation with oxLDL (oxidized LDL, Bioconnect, J65591), to mimic exposure of macrophages to modified lipoproteins in the atherosclerotic plaque.

In the in vitro experiment with cells of healthy donors, PBMC isolation was performed, cells were seeded in flat-bottom plates, washed, and cultured to monocyte-derived macrophages in 10% pooled serum obtained from PA patients or EHT controls (obtained from study NCT 01 978 132, executed in our hospital). On day 6, the monocyte-derived macrophages were stimulated with LPS or P3C for 24 hours, after which supernatants were stored for analysis.

### Measurement of inflammatory markers

Plasma E-selectin, matrix metalloproteinase (MMP)-2 vascular cell adhesion molecule (VCAM)-1 high-sensitivity C-reactive protein (hsCRP), IL-18, and IL-6 levels were determined with enzyme-linked immunosorbent assays (ELISAs). For E-selectin, MMP-2, VCAM-1, hsCRP, and IL-18, DuoSet ELISA (R&D Systems) was used. For measurement of circulating IL-6, high-sensitivity Quantikine ELISA assays (R&D) were used. Cytokine concentrations in PBMC/macrophage supernatants were measured by commercial ELISA kits according to the manufacturer’s instructions: TNF-α, IL-6, IL-8, and IL-10. IL-1β, IL-1 receptor antagonist, IL-17, and IL-22 (DuoSet ELISA, R&D), IFNγ (Sanquin).

### RNA isolation

CD14^pos^ MACS-isolated monocytes stored at baseline were isolated from TRIzol using a TRIzol/RNeasy hybrid protocol. In short, per 1 mL of TRIzol 200 μL of chloroform was added, mixed, incubated at room temperature for 5 minutes, and spun down for 15 minutes at 12 000 *g* at 4°C. The upper aqueous phase was transferred to a RNA-se free Eppendorf (Hamburg, Germany) tube, and an equal volume of 70% ethanol was added. After thorough mixing, the sample was loaded onto RNeasy mini columns (Qiagen), after which the manufacturer’s protocol was followed. After the last manufacturer’s step, 15 μL of RNase-free water was added to the columns, incubated for 5 minutes, and spun down.

### RNA sequencing and differential gene expression analysis

The concentration of RNA was determined on the Qubit; the quality using Nanodrop technology. Library preparation was performed using the Quantseq 3-inch messenger RNA (mRNA)-Seq Library Prep Kit-FWD from Lexogen (catalog No. 015.96, Lexogen) according to the manufacturer’s protocol. RNA input for all samples was normalized to 150 ng. All samples were processed in a single library preparation. After quality controls of each library was assessed using Qubit and tape station, libraries were pooled and diluted to 4 nM. Thereafter, sequencing of the libraries was performed on a NextSeq 500 instrument (Illumina) with a 1.4 pM final loading concentration; all libraries were sequenced in one sequencing round.

Low-quality filtering and adapter trimming was performed using Trim Galore!, V0.4.4_dev 9 (Babraham Bioinformatics), a wrapper tool around the tools Cutadapt version 1.18, and FastQC version 0.11.5 (Babraham Bioinformatics). Reads were mapped to a human reference genome (GRCh38.95, Ensembl) with Star v2.6.0a (22), resulting in BAM. These BAM files were counted (number of reads mapped to a feature, eg, a gene) with HTSeq (HTSeq-count tool version 0.11.0 (23)) using a complementary .gtf file, containing annotation for GRCh38.95 (Ensemle). MultiQC was used to combine results and quality checks of all the samples (24). Total reads were between 14 million and 17 million, of which percentage uniquely assigned reads were between 52% and 61%, aligned reads between 79% and 83%. LogFold shrinkage was performed with apeglm for easier comparison between groups (25). Differential gene expression analysis was carried out with DESeq2 version 1.22.0 in R (26), with the internal statistical and normalization method (ie, adjustment of *P* value for multiple testing with Benjamini-Hochberg). The average expression of the patient group vs control group was tested, with correction for sex. *P*-adjusted values of less than .05 were considered significant.

### Quantitative polymerase chain reaction

From patient-specific monocyte-derived macrophages, total mRNA for quantitative polymerase chain reaction (qPCR) was extracted using TRIzol (Life Technologies) according to the manufacturer’s protocol. iScript Reverse Transcriptase (Invitrogen) was used to synthesize complementary DNA. qPCR was performed on an Applied Biosciences StepOne PLUS qPCR machine using SYBR Green (Invitrogen), and the values expressed as log2-fold increase in mRNA levels in cells from PA patients relative to those in cells from EHT controls. *B2M* was used as a housekeeping gene.

### Statistical analysis

We analyzed the data with SPSS 22.0. Demographic data are presented as mean with SD when normally distributed or as median with minimum and maximum values for nonnormally distributed continuous variables, and as percentages for categorical data. Normality of all data was explored with histograms and the Shapiro-Wilk test. Differences in baseline characteristics were tested with an independent samples T test or a Mann-Whitney U test for continuous parameters and with the chi-square test in case of categorical data.

For PET data, we log-transformed the TBR prior to statistical analysis. Outliers (defined as z score <–2 or >2 after log transformation) were removed, which resulted in a maximal removal of 1 participant per ROI. Differences in log-transformed TBRs (or log-transformed SUV [liver]) between PA patients and EHT controls were tested using an analysis of covariance (ANCOVA) model to account for age (model 1) and additionally in a second ANCOVA model to account for age, and systolic and diastolic blood pressure (model 2).

Group differences in inflammatory and ex vivo cytokines were log-transformed before analysis with ANCOVA; age and sex were used as covariates.

All correlations were determined on nontransformed data using Spearman rank correlation and plotted with Loess curves. Relative gene expression values assessed with qPCR were transformed into Z scores and tested with ANCOVA corrected for age. All tests were 2-tailed.

*P* values of less than .05 were considered statistically significant. For RNAseq data, a *P*-adjusted value less than .05 was considered significant.

## Results

### Baseline characteristics

PA patients and EHT control patients were comparable in all baseline parameters (Table 1).

### Patients with primary aldosteronism show enhanced arterial wall inflammation and hematopoietic activity

**Figure 1.**
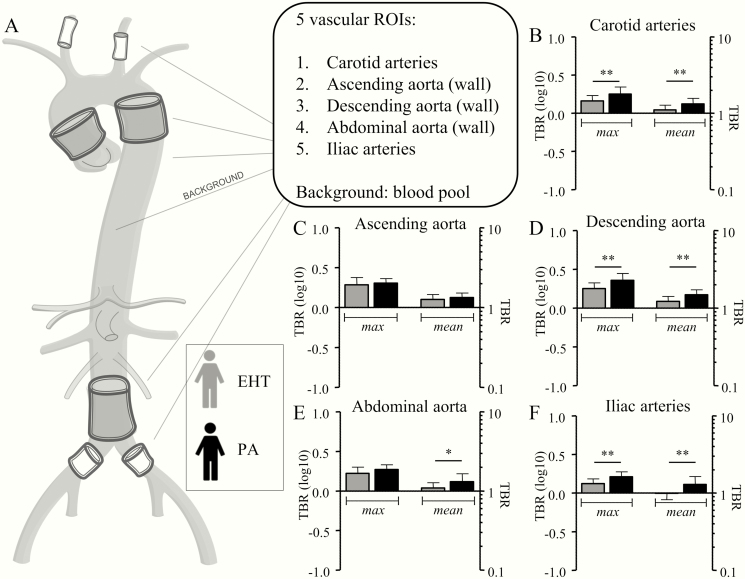
Patients with primary aldosteronism (PA) display enhanced vascular wall inflammation. A, Vascular inflammation was measured in 5 regions of interest (ROIs): the carotid arteries, the wall of the ascending, descending and abdominal aorta, and the iliac arteries. To calculate the target-to-background ratio (TBR), the blood pool in the low thoracic artery was used as background. B, In patients with PA, the carotid arteries have a significantly higher maximal and mean TBR. C, No differences in uptake were seen in the wall of the ascending aorta. D, In the wall of the descending aorta, mean and maximal TBR were significantly higher in patients with TBR; E, the same holds true for the mean TBR of the wall of the abdominal aorta, F, as well as for the mean and maximal TBR of the iliac arteries. TBR, target-to-background ratio. *P < .05; **P < .01.

**Figure 2.**
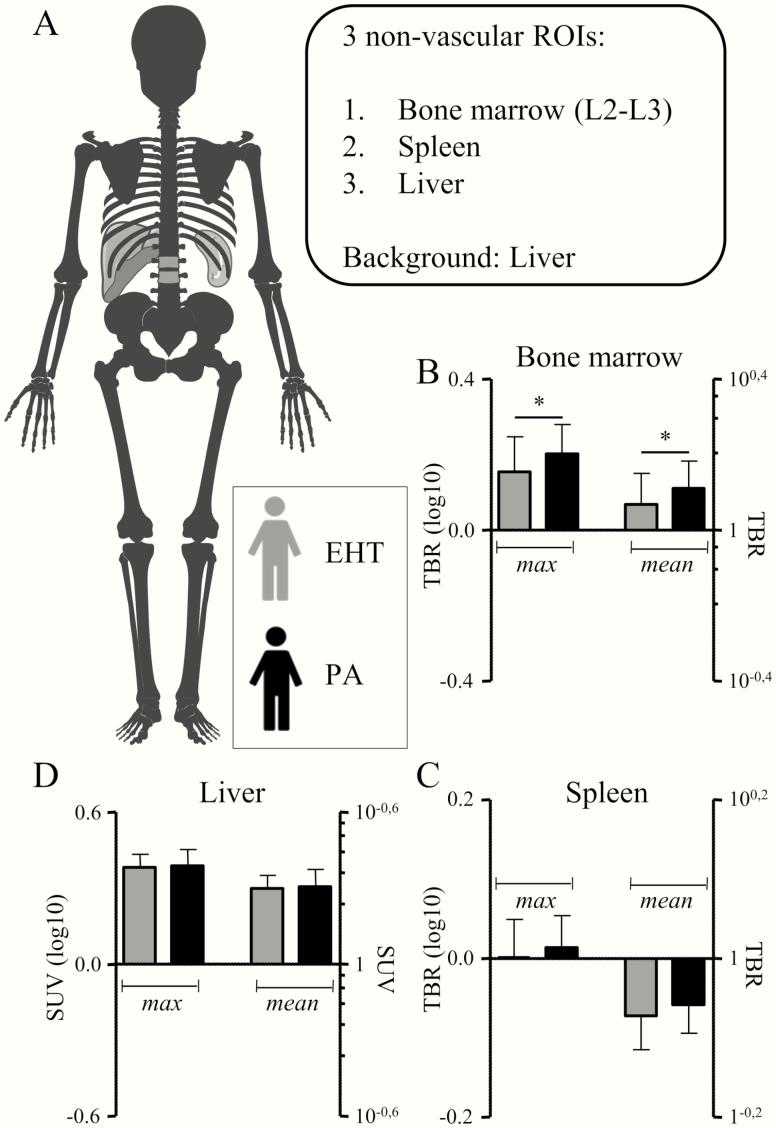
Patients with primary aldosteronism display increased hematopoietic activity. A, The ^18^FDG uptake was measured in 3 nonvascular regions of interest (ROIs): in the bone marrow (corpora L2 and L3) and spleen to determine hematopoietic activity, and in the liver. The ^18^F-FDG uptake in liver tissue was used as background. B, The TBR was higher in the bone marrow, C, but similar in the spleen. D, Also, liver SUV was comparable between patients and controls. TBR, target-to-background ratio. SUV, standardized uptake value. *P < .05.

**Figure 3.**
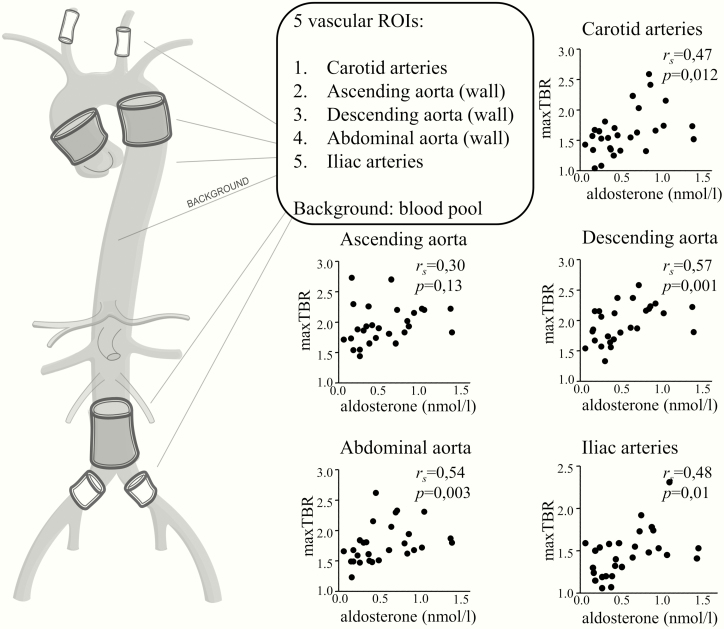
Aldosterone levels correlate with the extent of arterial wall inflammation. Vascular inflammation was measured in 5 regions of interest (ROIs): the carotid arteries, the wall of the ascending, descending and abdominal aorta, and the iliac arteries. To calculate the TBR, the blood pool in the low thoracic artery was used as background. Both in patients with PA as well as essential hypertension controls, aldosterone levels are correlated with the maximal TBR in all ROIs except for the ascending aorta. TBR, target-to-background ratio. *P < .05; **P < .01.

^18^F-FDG uptake was assessed at multiple vascular and hematopoietic ROIs and in the liver, as illustrated in Figs. 1A and 2A, respectively. In PA patients, the ^18^F-FDG uptake was higher in the carotid arteries, descending aorta, abdominal aorta, and iliac arteries, but not in the ascending aorta (data are depicted in Fig. 1B-1F, and Table 2). In line with this, aldosterone levels correlated with the mean and maximal TBRs of all vascular ROIs, except for the ascending aorta (Fig. 3). Also, ^18^F-FDG uptake in the bone marrow was significantly higher in PA patients (Table 2 and Fig. 2B). Because blood pressure was marginally higher in patients with PA (not reaching statistical significance), next to our intended model of analysis correcting for age (model 1), we present *P* values for an additional blood pressure–corrected model 2 in Table 2.

**Table 2.** Differences between PA patients and EHT controls in ^18^F-FDG uptake in vascular and nonvascular ROIs

ROI	EHT, Mean ±SD	PA, Mean ±SD	Model 1	Model 2
Ascending aorta (mean TBR)	1.26 ± 0.21	1.42 ± 0.33	*P* = .108	*P* = .144
Ascending aorta (max TBR)	1. 96 ± 0.46	2.23 ± 0.78	*P* = .266	*P* = .389
Descending aorta (mean TBR)	1.24 ± 0.19	1.51 ± 0.23	*P* = .004	*P* = .020
Descending aorta (max TBR)	1.82 ± 0.29	2.32 ± 0.50	*P* = .003	*P* = .011
Carotid arteries (mean TBR)	1.11 ± 0.15	1.53 ± 0.74	*P* = .006	*P* = .016
Carotid arteries (max TBR)	1.46 ± 0.22	2.16 ± 1.36	*P* = .007	*P* = .016
Abdominal aorta (mean TBR)	1.10 ± 0.16	1.34 ± 0.33	*P* = .033	*P* = .028
Abdominal aorta (max TBR)	1.69 ± 0.33	1.98 ± 0.46	*P* = .108	*P* = .256
Iliac artery (mean TBR)	1.00 ± 0.19	1.33 ± 0.35	*P* = .003	*P* = .010
Iliac artery (max TBR)	1.33 ± 0.19	1.76 ± 0.53	*P* = .001	*P* = .003
Corpora (mean TBR)	1.19 ± 0.22	1.31 ± 0.11	*P* = .014	*P* = .010
Corpora (max TBR)	1.45 ± 0.30	1.62 ± 0.29	*P* = .032	*P* = .042
Spleen (mean TBR)	0.85 ± 0.08	0.88 ± 0.07	*P* = .215	*P* = .876
Spleen (max TBR)	1.01 ± 0.11	1.04 ± 0.10	*P* = .351	*P* = .939
Liver (mean SUV)	2.00 ± 0.24	1.92 ± 0.57	*P* = .972	*P* = .698
Liver (max SUV)	2.42 ± 0.30	2.32 ± 0.67	*P* = .969	*P* = .680

Model 1: analysis of covariance model with covariate age.

Model 2: analysis of covariance model with covariates age, systolic blood pressure, and diastolic blood pressure.

Abbreviations: EHT, essential hypertension; max, maximum; PA, primary aldosteronism; ROI, region of interest; SUV, standardized uptake value; TBR, target-to-background ratio.

Splenic uptake and liver uptake were similar between PA patients and EHT control patients (Fig. 2C and 2D). Aldosterone levels did not correlate with these parameters (data not shown).

### Aldosterone levels correlate with the immune cell composition of peripheral blood

**Figure 4.**
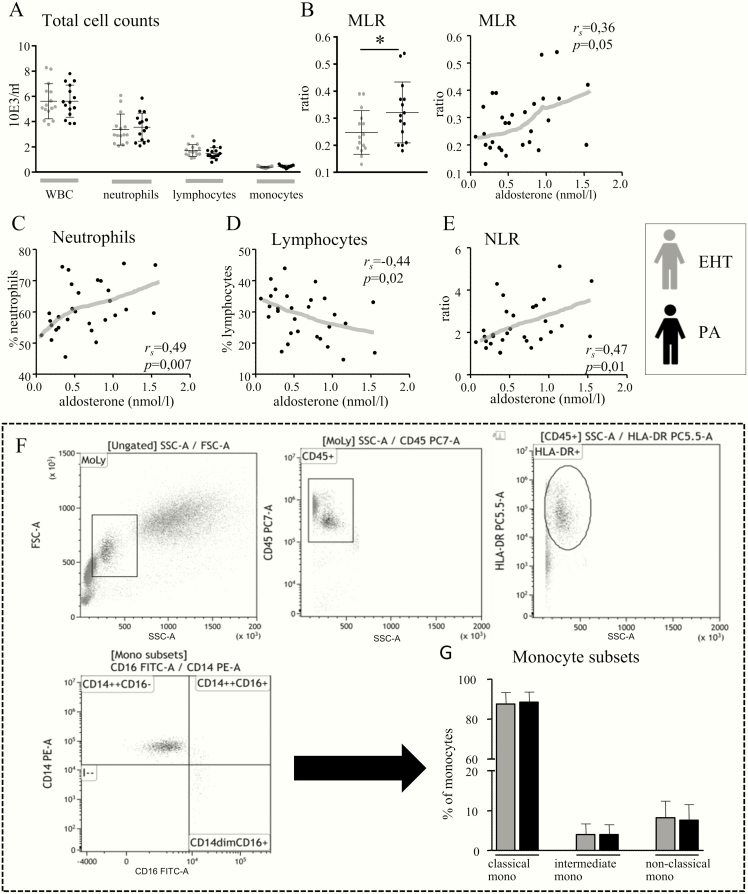
Peripheral blood cell composition in patients with primary aldosteronism (PA). A, Total cell counts in the peripheral blood did not differ between PA patients and essential hypertension controls. B, The MLR was higher in PA patients. Likewise, aldosterone levels showed a positive correlation with this ratio. C, Aldosterone levels correlated strongly with neutrophil percentages, D, showed a negative correlation with lymphocyte percentages, and E, showed a positive correlation with the NLR. F, Monocytes were further classified into subsets using flow cytometry. G, There were no differences in classical (CD14++/CD16–), intermediate (CD14++/CD16+) or nonclassical (CD14dim/CD16+) populations. MLR, monocyte-to-lymphocyte ratio; NLR, neutrophil-to lymphocyte ratio.

There were no differences in peripheral blood cell populations between PA patients and EHT patients (Fig. 4A and 4B). However, the inflammatory monocyte-to-lymphocyte ratio (MLR) was significantly higher in PA patients (0.32 ± 0.11 vs 0.25 ± 0.08, *P = *.047) (Fig. 4C), paralleling a trend toward a positive correlation between aldosterone and this ratio (Fig. 4D). Aldosterone correlated with relative neutrophil counts (Fig. 4E) and showed a negative correlation with relative lymphocyte counts (Fig. 4F). In line, it was correlated with the inflammatory neutrophil-to-lymphocyte ratio (NLR) (Fig. 4G). Monocyte subsets were similar between PA patients and EHT controls (Fig. 4H and 4I).

### Circulating markers of inflammation, endothelial dysfunction, and plaque instability are unchanged in primary hyperaldosteronism

**Figure 5.**
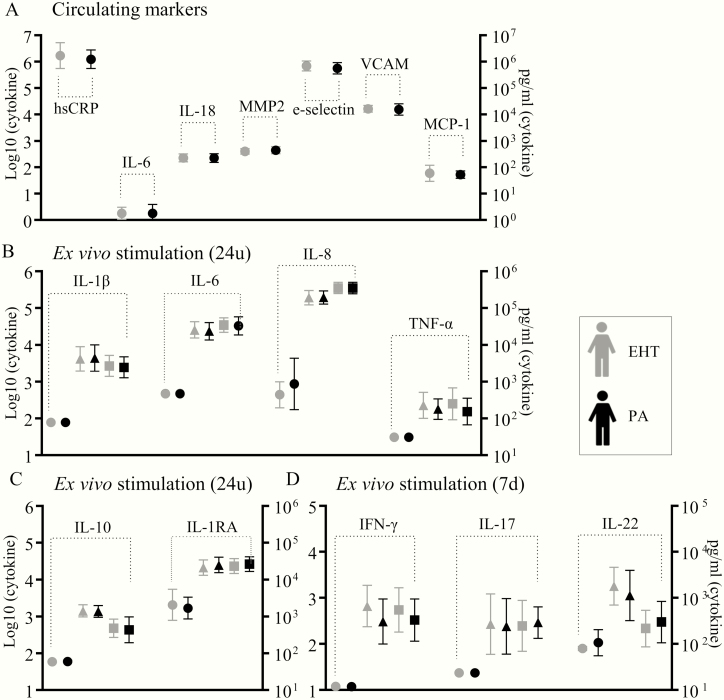
Circulating inflammatory markers and ex vivo cytokine production in primary aldosteronism (PA). A, No differences between PA patients (n = 15) and essential hypertension controls (n = 15) were observed in circulating parameters of inflammation (hsCRP, IL-6, IL-18, MCP-1), plaque instability (MMP2), and endothelial dysfunction (e-selectin, VCAM, MCP-1). Ex vivo PBMCs cytokine production was comparable for B, proinflammatory, and C, anti-inflammatory, monocyte-derived cytokines after 24 hours. D, There were no differences in lymphocyte-derived cytokines.

None of the tested circulating inflammatory markers, markers of endothelial dysfunction and matrix metalloproteinases differed between PA patients and EHT control patients (Fig. 5A).

### Ex vivo cytokine production is unchanged in primary hyperaldosteronism

There were no significant differences between PA patients and EHT control patients in ex vivo pro- or anti-inflammatory monocyte-derived cytokine production on stimulation (Fig. 5B and 5C). Also, lymphocyte-derived cytokine production was similar between both groups (Fig. 5D).

### The Monocyte transcriptome of primary aldosteronism patients is not suggestive of trained immunity

We performed RNA sequencing on CD14+ isolated monocytes from 4 PA patients and 4 age-matched and sex-matched EHT control patients. Using a cutoff of false discovery rate (FDR) < 0.05, 6 genes were differentially expressed: Five genes were significantly upregulated (*BTG2, WHAMMP3*, *HES1, HLA-A*, *FFAR2*), and 1 gene significantly downregulated (*HLA-DRB5*). This latter observation probably reflects allelic variation of HLA-DRB1/HLA-DRB5 between patients and controls. To increase sensitivity to detect potentially relevant transcriptomic changes despite our small sample size, we also explored additional signals with an FDR < 0.20 (Table 3), as has been described before (27). In line with the ex vivo stimulation data, none of these genes classically associates with inflammation or trained immunity (ie, genes encoding classical proinflammatory cytokines and chemokines or involved in glycolysis or cholesterol metabolism (28)). Instead, we identified upregulated genes associated with the regulation of apoptosis (*BTG2, IER3*, *PROK2*), immune defenses (*HLA-A*, *FCAR*), autophagy (*TP53INP2*), fatty acid metabolism (*FFAR2*), monocyte differentiation (*DUSP5*), (alternative) monocyte subset activation and immunosuppression (*HES1, JUNB*, *KLF2*), and monocyte migration (*PLAUR* [*CD87*]). Also, the transcriptionally regulator *EGR1,* important in induction of inflammatory responses on various stimuli, such as growth factors, cytokines, and oxidative stress, was among these genes.

**Table 3.** Transcriptome analysis reveals several genes expressed at FDR less than 0.20 (PA vs EHT)

Gene	Log2 Fold Change	FDR
*BTG2*	1.482 318	0.000 324
*WHAMMP3*	3.838 738	0.003 154
*HES1*	2.44 943	0.008 553
*HLA-DRB5*	–4.35 786	0.008 553
*HLA-A*	0.594 109	0.011 223
*FFAR2*	1.177 988	0.018 185
*JUNB*	0.942 873	0.097 491
*IER3*	0.826 127	0.097 491
*PROK2*	1.327 246	0.097 491
*CSRNP1*	0.931 404	0.097 491
*DUSP5*	1.386 709	0.097 491
*KLF2*	0.643 016	0.097 491
*SLC25A25*	1.148 992	0.097 491
*FCAR*	1.31 556	0.097 491
*PLAUR*	0.647 366	0.097 491
*CD83*	2.364 899	0.121 271
*HBA1*	3.827 395	0.138 236
*EGR1*	2.219 332	0.162 944
*G0S2*	2.736 997	0.162 944
*TP53INP2*	1.887 004	0.162 944
*TRIB1*	0.823 838	0.162 944
*OTUD1*	1.206 445	0.162 944
*QSER1*	–0.60 009	0.162 944
*RN7SL600P*	1.080 073	0.176 993
*Z83843,1*	–1.48 366	0.176 993

Abbreviations: EHT, essential hypertension; FDR, false discovery rate; PA, primary aldosteronism.

### Macrophages from patients with primary hyperaldosteronism express more *TNFA*

**Figure 6.**
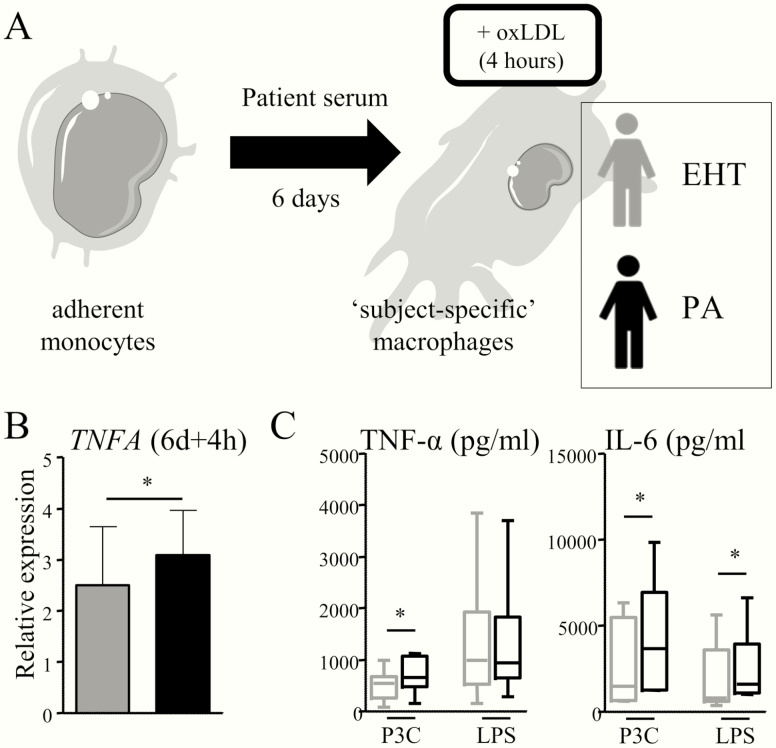
Macrophages cultured from primary aldosteronism (PA) patients are characterized by a higher expression of *TNFA.* A, Adherent monocytes from each study participant were differentiated to macrophages in medium enriched with the serum of that same individual, to culture “subject-specific” macrophages. B, On oxLDL stimulation, macrophages cultured from PA patients had higher *TNFA* expression than macrophages obtained from EHT controls. C, Next, adherent monocytes from healthy donors were differentiated to macrophages in the pooled serum of PA patients or the pooled serum of EHT controls for 6 days. On day 6, the cells were stimulated with P3C or LPS, and IL-6 and TNF-α production was measured. Both TNF-α production on P3C stimulation and IL-6 production on P3C and LPS stimulation were higher in cells cultured in the serum of PA patients. *P < .05.

Isolated monocytes obtained from each study participant were differentiated to macrophages in medium enriched with 10% serum from that same individual for 6 days, to obtain “subject-specific” macrophages (Fig. 6A). After 4 hours of oxLDL exposure, *TNFA* expression was significantly higher in macrophages cultured from PA patients (relative expression 3.1 ± 0.9 vs 2.5 ± 1.1. *P = *.01) (Fig. 6B). *IL6* expression followed the same trend (2.6 ± 2.3 vs 1.9 ± 3.1. *P = *.13).

To validate that the serum of PA patients can reprogram monocyte-derived macrophages, we performed an additional experiment. Adherent monocytes obtained from healthy donors (n = 6) were differentiated to macrophages in the pooled serum of PA patients or EHT patients. On day 6, cells were stimulated P3C and LPS. Cells cultured in PA serum showed an enhanced production of TNF-α on P3C stimulation and IL-6 on P3C as well as LPS stimulation (Fig. 6C).

We recently reported that activation of the fatty acid synthesis pathway contributes to aldosterone-induced trained immunity (8). We now tested whether the expression of genes in the fatty acid synthesis pathway (*ACACA*, *FASN*, and *ELOVL6)* in monocyte-derived macrophages was higher in PA patients, but there was no difference compared to EHT patients.

## Discussion

In this study, we show that PA is associated with low-grade arterial wall inflammation on ^18^F-FDG PET-CT. This is accompanied by an increased ^18^F-FDG uptake of the bone marrow, suggestive of enhanced hematopoietic activity. With these results, our study is the first to reveal a role for inflammation in patients with PA. However, this was not associated with inflammatory reprogramming of circulation monocytes.

Increased ^18^F-FDG uptake in the arterial wall reflects low-grade inflammation of the arterial wall, which predisposes to atherosclerosis, and which predicts future cardiovascular events (29). In histological studies, ^18^F-FDG uptake correlates with macrophage density in both animal and human atheromas (30). Our study shows that PA patients have an increased ^18^F-FDG uptake in various arterial regions compared to matched controls with essential hypertension, which is in line with animal studies previously showing a proatherogenic effect of aldosterone (6, 7). The linear correlation between aldosterone and vascular wall ^18^F-FDG uptake both in PA patients and EHT controls further suggests a causal relationship between aldosterone and arterial wall inflammation. The lack of differences in ^18^F-FDG uptake in the ascending aorta is likely explained by the high blood flow in this segment of the aorta, which makes this region less prone to the development of atherosclerosis (31).

Previous studies investigating atherosclerosis using ^18^F-FDG PET in high-risk populations observed a concomitant increased FDG uptake in hematopoietic organs. This reflects increased myeloid progenitor cell activation and proliferation, and is associated with an increased risk of future cardiovascular events (18, 32, 33). In line with these findings we also observed an increased activity of the bone marrow in PA patients compared to hypertensive controls. This coincided with changes in the peripheral blood cell composition, favoring myeloid cells over lymphocytes as reflected in the monocyte-to lymphocyte. This ratio, as well as the neutrophil-to-lymphocyte ratio, predicts poor outcomes in atherosclerotic diseases (34–37).

Although atherosclerosis is often associated with high inflammatory markers, we did not observe differences in circulating hsCRP and IL-6—among other markers of inflammation and endothelial dysfunction—between PA patients and EHT controls. Although one large population-based study showed a small, statistically significant difference in hsCRP between PA patients and EHT controls (1.6 mg/L vs 1.5 mg/L), others describe findings similar to ours (38). The absence of systemic inflammation highlights an important difference with previous murine work, in which renin–angiotensin–aldosterone system activation is generally reported to induce systemic inflammation (6). Importantly, next to differences in immunology between mice and humans, most murine models are hindered by blood pressure differences between intervention and control animals resulting from the sequential induction of hypertension by renin–angiotensin–aldosterone system activation. This might in part explain the differences between murine and human studies (6).

We recently showed that in vitro exposure of monocytes to aldosterone causes immunological imprinting, resulting in a proinflammatory macrophage phenotype after monocyte-to-macrophage differentiation. This “trained immunity” was dependent on the increase of fatty acid synthesis in aldosterone-trained macrophages (8). Against our expectations, circulating monocytes of PA patients did not show an inflammatory footprint suggestive of in vivo training in terms of an enhanced cytokine production capacity (14). Also, we did not observe increased activity of the fatty acid synthesis pathway in the transcriptome of circulating monocytes, nor in macrophages cultured from PA patients. Because ex vivo immune responses were previously shown to be dysregulated in EHT patients compared to normotensive volunteers, and to normalize on normalization of blood pressure, it is possible that compared to healthy individuals, circulating monocytes of PA patients are phenotypically different (39, 40). Only one study previously reported on ex vivo responsiveness of leukocytes in PA. In this study, 4 patients with PA were compared with 10 sex-, age-, and blood pressure matched controls; a higher proinflammatory cytokine production was observed in PA patients. Importantly, the difference in aldosterone levels between PA patients in this cohort (1.3-2.0 nmol/L) and controls (on average 0.26 nmol/L) is much larger than in our study, which might explain the contrasting findings. Moreover, information on medication use in the essential hypertensive subgroup was lacking; various antihypertensives as well as statins have immunomodulatory effects and could have affected the results. Last, in recent years the cosecretion of glucocorticoids in PA has gained attention. In a recent large cohort trial of more than 200 patients with PA, the excretion of glucocorticoid metabolites was shown to be comparable to patients with subclinical Cushing disease. Glucocorticoids are known to exert various anti-inflammatory actions (41), among which are inhibition of TLR-downstream signaling, attenuation of chemokine cascades and leukocyte migration, and reprogramming of macrophages to an alternative, anti-inflammatory subtype. Given these actions, subtle glucocorticoid excess that coexists next to aldosterone excess in PA might suppress inflammatory skewing of leukocytes by aldosterone itself. The lack of this glucocorticoid excess in preclinical work could explain the discrepancy with both our previous work (8) as well as others’ (6).

RNA sequencing confirmed that the transcriptome of PA patients and EHT control patients does not show changes classically associated with trained immunity (ie, upregulation of genes encoding proinflammatory cytokines or regulating glycolysis, cholesterol, or fatty acid metabolism (28)). However, in the top upregulated genes in PA patients compared to EHT controls, several genes associated both with induction as well as suppression of inflammation, monocyte differentiation, trafficking, and apoptosis were found. Interestingly, several of these genes have previously been suggested to be associated with cardiovascular pathology (*FCAR* (42), *PLAUR* (43), *EGR1* (44)), whereas others have been implied to be protective (*KLF2* [45]). Several genes were previously shown upregulated in circulating leukocytes in patients with peripheral artery disease (*FCAR*, *FFAR2, DUSP5*, *PLAUR)* (46). Importantly, because of our small sample size, we used an exploratory approach analyzing genes with an FDR < 0.20, and these findings need further validation.

It is important to realize that ^18^F-FDG-uptake in the arterial wall reflects an increase in the net local glucose metabolism (to which all metabolically active cell types in the vasculature contribute) rather than conferring monocyte specificity alone. Both increased glycolytic capacity of one or more cell populations, or an increase in the total number of metabolically active cells, could on theoretical grounds equally contribute to an in increased ^18^F-FDG signal. Therefore, there are several mechanisms that could explain our finding of increased ^18^F-FDG-uptake in the arterial wall of patients with PA, despite only modest changes in the circulating leukocyte composition and monocyte phenotype. First, we observed a higher *TNFA* expression in macrophages cultured from PA patients exposed to oxLDL. With this model, we aimed to mimic the inflammatory environment in the vascular wall, where macrophages are the main inflammatory effector cells, and abundantly exposed to oxLDL particles. In addition, macrophages obtained from healthy donors produced more proinflammatory cytokines when cultured in the serum of PA patients compared to EHT control patients. These data drive the hypothesis that monocyte-to-macrophage differentiation is necessary to unveil pro-inflammatory changes in the presence of hyperaldosteronism. Alternatively, macrophages in the atherosclerotic plaque not only derive from circulating monocytes, but in part develop from proliferating tissue-resident macrophages. Fate mapping studies revealed that these cells originate from the fetal liver before birth, or are recruited from blood shortly after birth, after which they become independent of the circulating pool of monocytes. Consequently, their phenotype differs from monocyte-derived macrophages (47). The effects of chronic exposure of (tissue-resident) macrophages to high aldosterone levels would importantly contribute to our understanding of vascular inflammation in PA. Second, next to macrophages, vascular smooth muscle cells (VSMCs) are the most abundant cells in the atherosclerotic plaque. Whereas VSMC proliferation is classically seen beneficial for plaque stability, in recent years numerous lines of evidence showed that VSMCs may undergo phenotypic switches in the atherosclerotic plaque, and that VSMC-derived macrophage-like cells promote local inflammation. Moreover, they importantly contribute to foam cell populations (48). Interestingly, VSCMs have been recently suggested to contribute to vascular wall ^18^F-FDG uptake (49, 50). Aldosterone was previously reported to induce inflammation in VSMCs, although a recent *ApoE* knockout model did not confirm a significant role for the smooth muscle cell MR in atherosclerosis (51). Third, endothelial cell activation might importantly contribute to monocyte recruitment to the vascular wall, and subsequently to macrophage accumulation in the developing plaque (52). Previously, the mineralocorticoid receptor was already shown to induce endothelial cell adhesion molecules expression, among other actions promoting inflammation (53).

In conclusion, our data suggest that aldosterone induces vascular wall inflammation in vivo in PA patients. In addition, we observed enhanced hematopoietic activity, and inflammatory changes in the leukocyte composition. However, we did not observe major differences in circulating innate immune cell phenotype. Therefore, it is most likely that interaction between various cell types culminates in significant arterial inflammation in PA. Future research should investigate the potential to reverse this local inflammation with MR antagonists or curative adrenalectomy. For the clinician, our data emphasize the importance of timely recognition of PA in the hypertensive population, and adequate treatment of all CVD risk factors present in these patients.

## Abbreviations

^18^F-FDG: 2’-deoxy-2’-(18F)fluoro-D-glucose
CT: computed tomography
CVD: cardiovascular disease
EHT: essential hypertension
MR: mineralocorticoid receptor
PA: primary aldosteronism
PET: positron emission tomography
TBR: target-to-background-ratio

## Notes

***Financial Support:*** N.R., L.J., and M.N. received funding from the European Union Horizon 2020 research and innovation program REPROGRAM under grant agreement No. 667837, and received an IN-CONTROL CVON grant from the Dutch Heart Foundation (CVON2012-03 and CVON2018-27). M.N. is further supported by a Netherlands Organization for Scientific Research Spinoza Grant (NWO SPI 94–212). L.J. is supported by a Competitiveness Operational Programme grant of the Romanian Ministry of European Funds (HINT, ID P_37_762; MySMIS 103587). N.R. is recipient of a grant from the ERA-CVD Joint Transnational Call 2018, which is supported by the Dutch Heart Foundation (JTC2018, project MEMORY; 2018T093). J.D. is supported by the Deutsche Forschungsgemeinschaft (CRC/TRR 205).

## Additional Information

***Disclosure Summary:*** C.H., E.S., E.A., M.N., H.M., S.K., C.K., A.H., J.D., L.J., M.N., N.R., have no financial interest to declare.

***Data Availability:*** The datasets generated during and/or analyzed during the current study are not publicly available but are available from the corresponding author on reasonable request.
